# A frequency and beamwidth reconfigurable antenna based on liquid crystal for 5G millimeter waves

**DOI:** 10.1038/s41598-025-01673-0

**Published:** 2025-05-22

**Authors:** Peng Chen, Xinju Wang, Dan Wang, Zongsheng Gan, Yutong Yin, Haowei Zhang

**Affiliations:** 1 School of Ocean Information Engineering, Jimei University, Xiamen, 361021 China; 2 Fujian Provincial Key Laboratory of Maritime Communication and Intelligent Electronic Systems, Jimei University, Xiamen, 361021 China; 3 Fujian Provincial Key Laboratory of Oceanic Information Perception and Intelligent Processing, Jimei University, Xiamen, 361021 China; 4 Tianfu Xinglong Lake Laboratory, Instrument Platform Department, Chengdu, 610200 China

**Keywords:** Electrical and electronic engineering, Design, synthesis and processing

## Abstract

In this paper, a novel frequency and beamwidth reconfigurable antenna is proposed. The antenna features a 5-layer vertically stacked structure with the dimensions of $22\times 30\times 7.768$ $\hbox {mm}^{3}$, and the layers from top to bottom are the radiating layer, the orientation layer, the liquid crystal (LC) layer, the orientation layer, and the ground layer, and a liquid crystal cavity integrated into the LC layer. An inverted microstrip feed line structure is employed as the bias electrode, and connecting it to a coaxial line side-feed adapter for excitation of the antenna. To investigate the beamwidth reconfigurability, two parasitic dipole structures-all disconnected or connected-are placed on either side of the main radiating element for comparative analysis. Experimental results reveal that the antenna’s resonance frequency shifts from 31.78 GHz to 27.1 GHz, providing a frequency reconfigurable range of 14.73%. Notably, this frequency tuning process is minimally influenced by the type of parasitic patch. Additionally, the impedance bandwidth and -3dB beamwidth of the antenna remain largely unaffected by the reconfiguration. Testing the antenna with different parasitic patch structures, the -3dB beamwidth of the antenna expands from $47^{\circ }$ to $92^{\circ }$ at $\varepsilon _r = 2.7$ for the LC, and from $53.5^{\circ }$ to $81^{\circ }$ at $\varepsilon _r = 3.1$, and the antenna peak gain of 6.04 dBi and 7.58 dBi, separately. These results correspond to a reconfigurable range of 64.75% and 40.89% for the -3dB beamwidth, respectively.

## Introduction

The application of millimeter-wave (mmWave) bands is a new frontier in wireless communications technology, promising seamless interconnection between ultra-high-speed wired networks and personal wireless devices^1^. The development of mmWave bands, which offer enormous bandwidth, will lead to a variety of new wireless products and services for the Internet of Things (IoT) and fifth generation (5G) mobile systems^2^. mmWave communication has become the focus of research within the framework of 5G communication technologies. This is primarily due to its significant potential in optimizing spectrum resource utilization^3,4^. Reconfigurable antennas exhibit a high degree of flexibility and functionality due to their ability to dynamically adjust resonant frequencies, bandwidths, polarization states, and radiation patterns^5^. These frequency and beamwidth reconfigurable antennas are particularly valuable for 5G applications, offering a broad range of frequency bands and effectively mitigating multipath fading, thereby enhancing the signal-to-noise ratio^6^. To meet the growing demand for higher frequency services, many countries and regions around the world have integrated millimeter-wave bands into the high-frequency resources of their 5G communication systems.Beamwidth is one of the most important characteristics of antenna radiation, which determines the signal coverage at a specific distance. Different applications usually require different beamwidths^7^. To achieve reconfigurability, current antenna designs often integrate various components, including p-i-n diode^8–10^, varactor diode^11,12^, MEMS switch^13,14^, or LC material^15,16^. In Ref.^17^, a pattern reconfigurable triangular patch array is presented, capable of operating across different tunable frequencies. The antenna’s resonant frequency can be adjusted between 1.97 GHz and 2.54 GHz, yielding a 25.3% tunable bandwidth by varying the capacitance of six varactor diodes (from 4.27 pF to 1.15 pF). In Ref.^18^, a microstrip patch antenna with pattern reconfigurable is proposed that utilizes liquid metal to modulate different beam angles, allowing the beam to be switched between multiple directions ($0^{\circ }$, $20^{\circ }$, $40^{\circ }$). Similarly, an LC-based frequency reconfigurable microstrip patch antenna is proposed in Ref.^19^, where liquid crystals (LCs) are used as the tuning material, solving the size constraints associated with traditional solid-state tuning elements. This design achieves a three-fold bandwidth enhancement at a center frequency of 30.3 GHz. Although a few antennas have implemented either frequency or beamwidth reconfigurable features in 5G communication environments, many designs still face limitations in their range of use^20^. Notably, only a few published reports have been able to achieve both reconfigurable features simultaneously. Even when one reconfigurable feature is realized, it often negatively impacts other performance parameters, limiting the overall effectiveness of the antenna.

For the antenna proposed in this paper offers a significant development in achieving dual reconfigurable modes, the five-layer structure is used in order to form a good orientation of the liquid crystal molecules while being able to minimize the mutual coupling between the two reconfigurable properties. In addition, the multilayer stacking structure can form a broadened bimodal or multimodal band by adjusting the dielectric constants of LCs in the LC layer to form two or more resonant frequencies that are close to each other. At the same time, slotting the microstrip patch to change the current path length on the antenna surface and adding parasitic patch structures are equivalent to introducing new resonant loops to realize bandwidth expansion. Considering that the parasitic effects during the conduction and cutoff of a conventional p-i-n diode can have an unpredictable impact on the radiation characteristics. Specifically, p-i-n diodes do not behave as ideal switches, i.e., as perfect short-circuits or open-circuits. To mitigate these parasitic effects-such as parasitic capacitance and inductance-the proposed design introduces a gap between the two arms of the parasitic patch, approximating the ideal on / off states and reducing their influence on antenna performance. The impedance bandwidth of the antenna is optimized by placing parasitic patches with the same structure on both sides of the main radiating patch, enhancing the overall performance. By adjusting the dielectric constant of the LC, the antenna is able to achieve resonant frequency modulation performance while greatly reducing the coupling effect between the two reconfigurable features. The integration of the LC with various parasitic patches enables continuous switching between 5G millimeter-wave bands in different countries and regions, including the U.S. (27.25-28.35 GHz), Japan (27.5-29.5 GHz), South Korea (26.5-29.5 GHz), and the European Union (31.8-33.4 GHz). Additionally, this design supports the reconstruction of a wide range of radiation bandwidths across different frequencies, providing flexibility and adaptability for diverse communication environments.

The paper is organized as follows. The ”Introduction” section introduces some information about the research of two different reconfigurable features in antennas. The ”Antenna design and mechanism analysis” section introduces the specific structural parameters of the proposed antenna and the realization mechanism of the two reconfigurable functions. The performance of the antenna is studied in depth in the ”Simulation and measurement results” section, and the simulation is verified under the actual test results. Finally, the ”Conclusion” section briefly summarizes the main contributions of the article and draws conclusions.

## Antenna design and mechanism analysis

### Antenna structure design

**Fig. 1:**
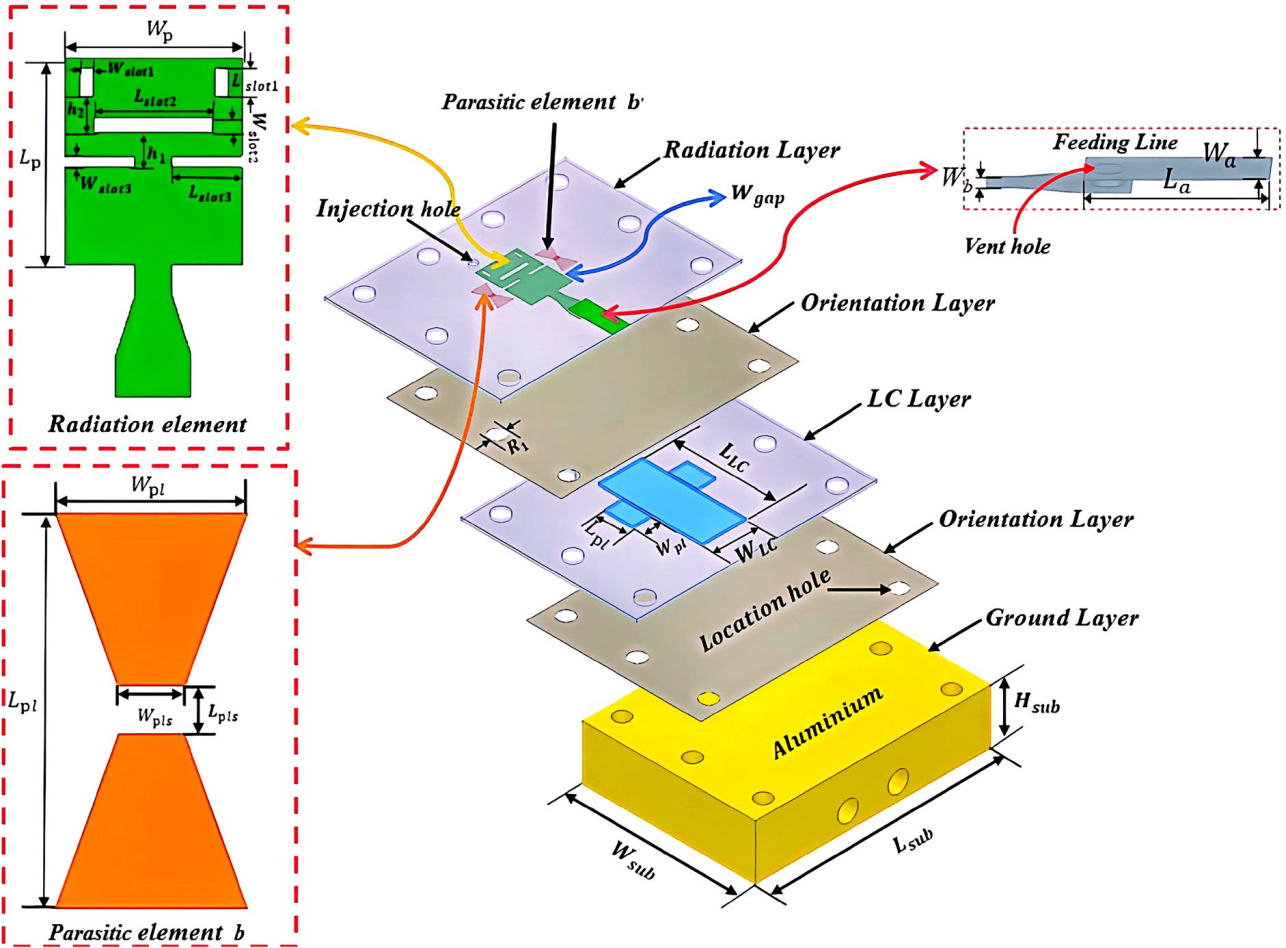
Exploded view and dimension parameters of the proposed antenna involving LC.

Figure 1 illustrates the geometry and related parameters of the proposed antenna. The antenna features a 5-layer stacked structure with a total size of $22\times 30\times 7.768$ $\hbox {mm}^{3}$. The primary radiating structure of the antenna consists of the main radiating patch with an H-shaped slot etched in the central, flanked by two parasitic patches. The parasitic patches, labeled *b* and *b*$'$, are identical in structure, with etched slits characterized by widths $W_{pls}$ and lengths $L_{pls}$, respectively. Its top vertical aperture and inverted microstrip line combine to form a side-fed structure that feeds the main radiating patch through a coaxial line.

**Table 1:** Dimension parameters and their values for the proposed antenna. (Unit: mm).

Radiation Layer													
$L_{p}$	7	$W_{p}$	5.2	$L_{a}$	5.5	$W_{a}$	2.2	$L_{pl}$	3.2	$W_{pl}$	2	$W_{b}$	1.1
$L_{slot1}$	1	$W_{slot1}$	0.4	$L_{slot2}$	3.5	$W_{slot2}$	0.5	$L_{slot3}$	2.05	$W_{slot3}$	0.4	$h_{1}$	1.2
$h_{2}$	1.2	$H_{r}$	0.381	$W_{gap}$	0.25	$W_{pls}$	0.6	$L_{pls}$	0.4				

Orientation Layer													
$R_{1}$	2	$H_{oren}$	0.03										

LC Layer													
$L_{LC}$	13	$W_{LC}$	8.7	$L_{pl}$	3.2	$W_{pl}$	2						

Ground Layer													
$L_{sub}$	30	$W_{sub}$	22	$H_{sub}$	7								

The antenna has a total of 5 layers. The first and third layers utilize Rogers RT/Duroid 5880 material as the dielectric substrate, which has a relative permittivity of 2.2 and a loss angle tangent of 0.0009. Both layers have a thickness of $H_{r}$. The first substrate has two through holes with a diameter of 1 mm, one for venting and the other for injecting LCs. The main radiating patch and the parasitic patch both have an edge distance of $W_{gap}$ and both are made of copper with a thickness of 35um. The third layer is a slot structure designed to accommodate the LCs. On the other hand, the second and fourth layers are orientation layers fabricated by spin-coating, curing, and rubbing processes using a polyimide solution having a dielectric constant of 3.4 and a loss angle tangent of 0.004. Their primary function is to preorient the LC’s molecules. The thickness of these layers is denoted as $H_{oren}$. The fifth layer consists of an aluminum block with a thickness of $H_{sub}$. It is flanked by two flange through-holes for securing the RF connector. Table 1 presents the optimized antenna parameters.

### Properties of liquid crystal materials

**Fig. 2:**
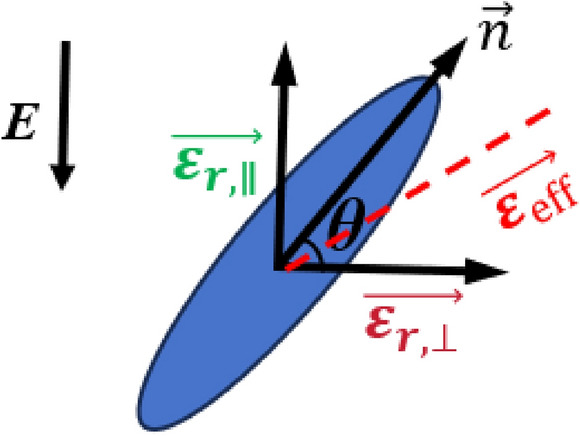
Schematic diagram of the LC molecule’s deflection direction.

Liquid crystal materials are intermediate between liquids and solids, having both liquid fluidity and crystalline anisotropy^19^. This research focuses on thermotropic LCs, in which the alignment of molecules can be controlled by external bias voltages, thereby modulating the dielectric tensor in various deflected states^21^. In the nematic phase of LCs, the molecules’ long axes are aligned nearly parallel to one another, though they do not exhibit a distinct layered structure. Under the influence of an electric or magnetic field, the molecules are free to reorient, leading to changes in the LC’s dielectric constant. Therefore, in this design, the LC of nematic phase is selected as the tuning material. As shown in Fig. 2, there is a schematic diagram of the deflection of nematic phase’s LC rod-like molecules with the long axis pointing to the vector $\vec {n}$ under an applied electric field^19^. Where *E* is the electric field direction of the applied bias voltage. $\varepsilon _{eff}$ represents the effective dielectric constant. The different angles of rotation of the rod molecules at different externally controlled bias voltages characterize the different dielectric constants of the liquid LCs, and $\vec {n}$ determines the tensor of dielectric constants in different deflection states, $\theta$ is the angle at which the liquid crystal molecules are deflected.

When $\vec {n} = (\cos {\theta }, 0, \sin {\theta })$, the relative dielectric constant tensor can be represented^19^:

1 $$\begin{aligned} \overleftrightarrow {\varepsilon _r} = \begin{pmatrix}\varepsilon _{r,\perp }+\Delta \varepsilon _r\cos ^2\theta & 0& \Delta \varepsilon _r\sin \theta \cos \theta \\ 0& \varepsilon _{r,\perp }& 0\\ \Delta \varepsilon _r\sin \theta \cos \theta & 0& \varepsilon _{r,\perp }+\Delta \varepsilon _r\sin ^2\theta \end{pmatrix} \end{aligned}$$

When no bias voltage is loaded, $\vec {n}$ is perpendicular to the direction of the electric field, $\theta$ is approximately $0^{\circ }$, and the relative dielectric tensor matrix of the LC is:

2 $$\begin{aligned} \overleftrightarrow {\varepsilon _{r,\perp }} = \begin{pmatrix} \varepsilon _{r,\parallel }& 0& 0\\ 0& \varepsilon _{r,\perp }& 0\\ 0& 0& \varepsilon _{r,\perp } \end{pmatrix} \end{aligned}$$

When the bias voltage loaded is greater than $\varepsilon _{eff}$, $\vec {n}$ is parallel to the direction of the electric field, $\theta$ is approximately $90^{\circ }$, then:

3 $$\begin{aligned} \overleftrightarrow {\varepsilon _{r,\parallel }} = \begin{pmatrix}\varepsilon _{r,\perp }& 0& 0\\ 0& \varepsilon _{r,\perp }& 0\\ 0& 0& \varepsilon _{r,\parallel }\end{pmatrix} \end{aligned}$$

The relative electrical tuning ability of nematic LC materials is:

4 $$\begin{aligned} \tau =\frac{\varepsilon _{r,\Vert }-\varepsilon _{r,\perp }}{\varepsilon _{r,\Vert }} \end{aligned}$$

Where $\varepsilon _{r,\perp }$ and $\varepsilon _{r,\parallel }$ are the relative permittivity of the LC’s molecule when its long axis is perpendicular and parallel to the biased electric field, respectively. The dielectric anisotropy of LC is $\Delta \varepsilon _r = \varepsilon _{r,\parallel } - \varepsilon _{r,\perp }$.

In general, the dielectric constant of LCs decreases at high temperatures and increases at low temperatures. This is due to the fact that the orientation of liquid crystal molecules is affected by temperature, and the arrangement of them are more disordered at high temperatures, which causes the intermolecular interactions to change, thus affecting the dielectric properties of LC. In addition, the loss factor ($\tan {\delta }$) of LC decreases at low temperatures and increases at high temperatures. Hence, drastic changes in temperature can cause liquid crystal materials to negatively affect the antenna’s tuning characteristics, impedance matching, and bandwidth. The positively oriented column-phase liquid crystal of model LC-BYIPS-P01 provided by Shanghai Hengshang Precision Instrument Co., Ltd. is used in this design, which is less sensitive to temperature. The properties of this liquid crystal material at room temperature are shown as follows: $\varepsilon _{r,\perp } = 2.2$, $\varepsilon _{r,\parallel } = 5.5$, and loss tangents of $\tan {\delta _{\perp }} = 0.0304$ and $\tan {\delta _{\parallel }} = 0.0146$.

### Frequency and beamwidth reconfigurable mechanism

The antenna comprises a main radiating microstrip patch, with the aluminum block as the positive and negative bias electrodes, respectively. By varying the potential difference loaded between the two electrodes, an electric field is generated within the LC cavity, which in turn deflect the LC’s molecules to change the dielectric constant of the LC, thereby affecting the wavelength of electromagnetic wave propagation within the medium and, consequently, tuning the antenna’s resonance frequency. In addition, an H-shaped slot is etched on the main radiating microstrip patch of the antenna, and by modulating the LC’s relative dielectric constant, the surface current distribution on the radiating patch is effectively modified, which is helpful for realizing the optimal impedance matching within the target passband while having similar radiating impedances, and facilitating the formation of a multi-resonance structure in order to improve the impedance bandwidth of the antenna. The antenna is designed based on the principles of the Yagi antenna, utilizing the main radiating patch and two parasitic dipole elements to optimize the overall radiation characteristics. The reconfigurability of the antenna’s -3dB beamwidth is achieved by controlling the slotted or unslotted configuration of the parasitic patches. This configuration directly influences the magnitude and phase distribution of the surface currents on the antenna, thereby adjusting the radiation pattern. When the parasitic patches are unslotted, since the total length of the parasitic patch on each side is slightly longer than half the wavelength, functions similarly to a reflector in a Yagi antenna, conversely, each parasitic patch can be regarded as a pilot. When the main driver element is excited, its voltage is $V_{2}$. Due to the existence of mutual coupling effect, parasitic elements *b* and *b*$'$ will generate induced voltages $V_{1}$ and $V_{3}$, respectively. Therefore, when constructing a three-port network, it is necessary to ensure that the voltage and current of the network satisfy the following relationship:

5 $$\begin{aligned} {[}V] = [Z][I] \end{aligned}$$

where,

6 $$\begin{aligned} {[}V]= & \begin{bmatrix}V_1\\ V_2\\ V_3\end{bmatrix} \end{aligned}$$

7 $$\begin{aligned} {[}Z]= & \begin{bmatrix}Z_{11}& Z_{12}& Z_{13}\\ Z_{21}& Z_{22}& Z_{23}\\ Z_{31}& Z_{32}& Z_{33}\end{bmatrix} \end{aligned}$$

8 $$\begin{aligned} {[}I]= & \begin{bmatrix}0\\ I_2\\ 0\end{bmatrix} \end{aligned}$$

As can be seen from Eq. (8), the ratio of the induced voltage on the two parasitic patches to the voltage on the main driver element is as follows:

9 $$\begin{aligned} \frac{V_{1}}{V_{2}}=\frac{Z_{12}}{Z_{22}} ,\quad \frac{V_{3}}{V_{2}}=\frac{Z_{32}}{Z_{22}} \end{aligned}$$

where $Z_{12}$ and $Z_{32}$ are the mutual between impedances parasitic patches *b* and *b*$'$ and main driving element 2, respectively, and $Z_{22}$ is the self impedance of the latter. The full-wave simulation of the antenna’s three patch design feed ports is carried out by electromagnetic simulation software HFSS, and after the reflection coefficient and transmission coefficient are obtained, the mutual impedance between the three microstrip patches can be extracted by port analysis. The self-impedance change of each patch at different frequencies can be visualized by using the impedance plot of HFSS.

By applying the aforementioned equation, the ratio between the induced voltage on the two parasitic elements and the voltage on the main drive element is obtained. This ratio reflects the degree of response of the parasitic units to the electromagnetic field of the main driving element. According to Eq. (9), an expression for the pattern of the reconfigurable antenna with parasitic patches can be obtained:

10 $$\begin{aligned} \begin{aligned} A_{(\theta )}&=V_{2}A_{2}(\theta )\left( \frac{V_{1}}{V_{2}}e^{-j\phi }+1+\frac{V_{3}}{V_{2}}e^{j\phi }\right) \\&=V_{2}A_{2}(\theta )\left( \frac{Z_{12}}{Z_{22}}e^{-j\phi }+1+\frac{Z_{32}}{Z_{22}}e^{j\phi }\right) \end{aligned} \end{aligned}$$

where $\phi = k_0 d\sin {\theta }$, $k_0$ is the propagation constant in free space, and $A_2 (\theta )$ is the radiation pattern of the antenna’s main driving element.

## Simulation and measurement results

**Fig. 3:**
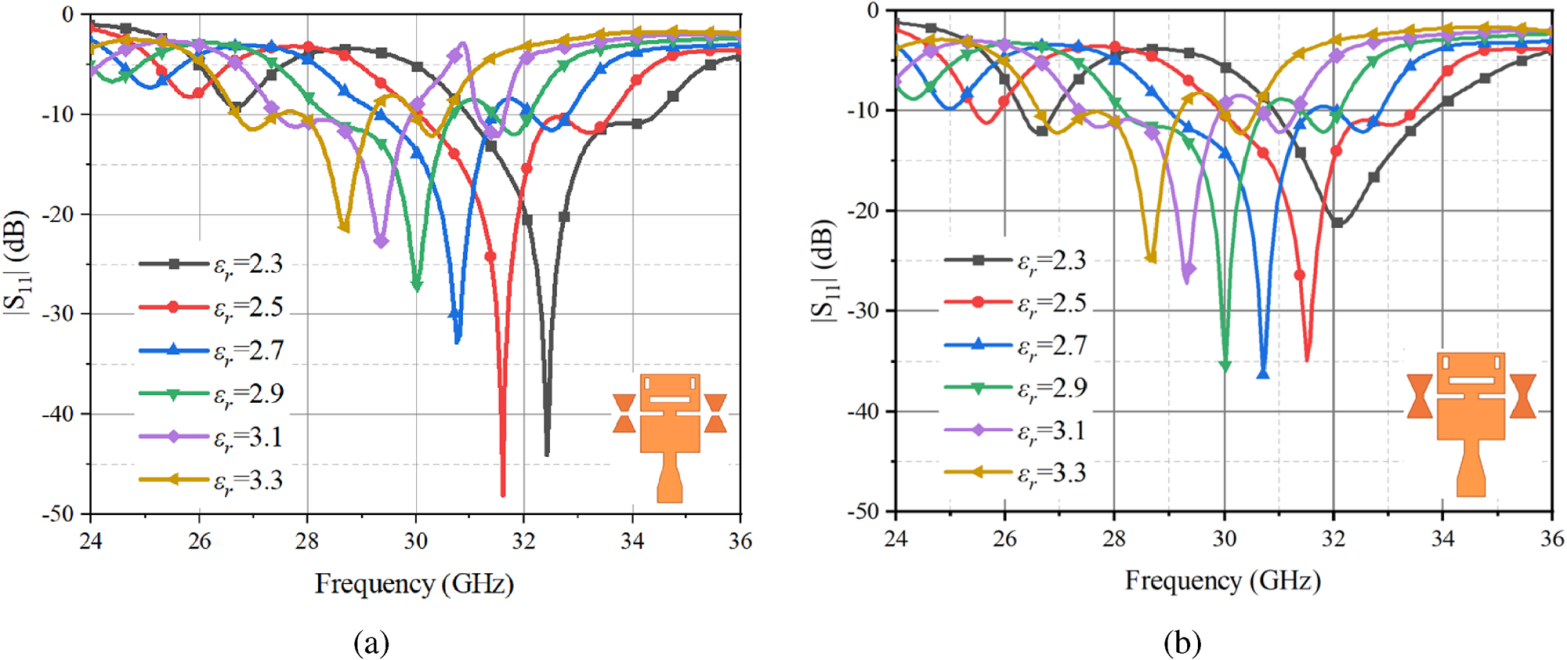
Simulation results of $|S_{11}|$ of two parasitic patches in (**a**) the cut-off state, and (**b**) the conductive state.

**Fig. 4:**
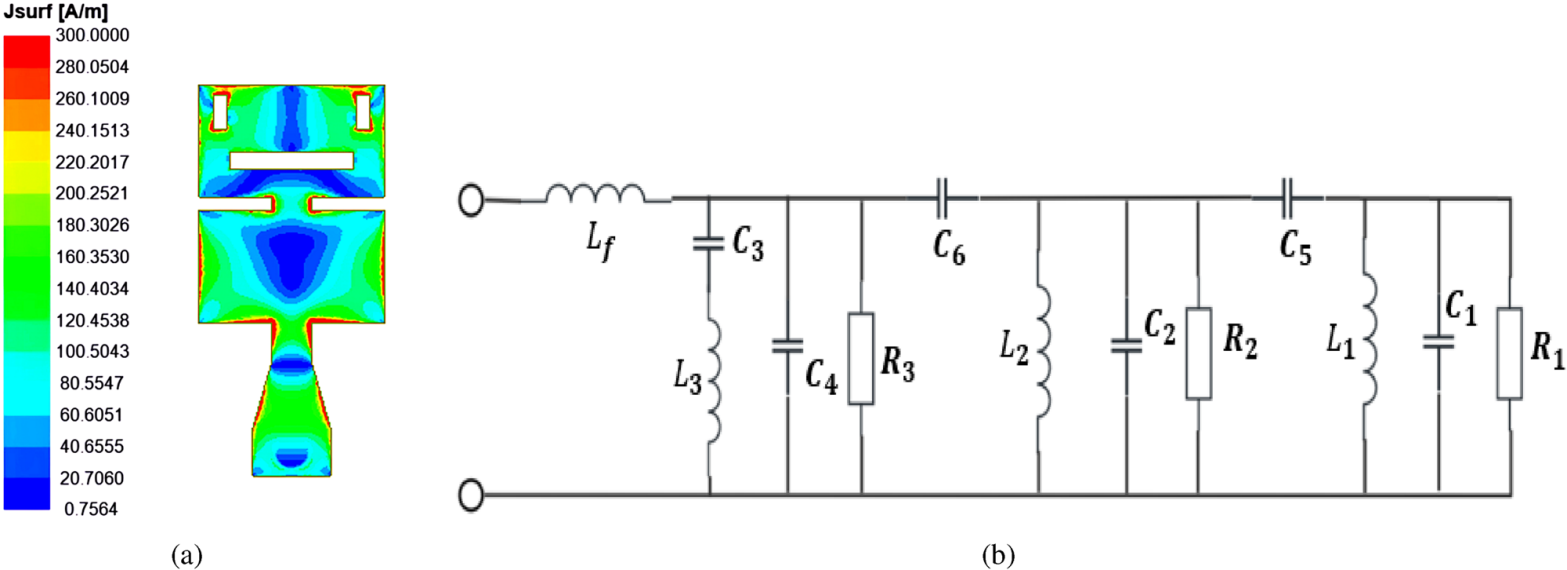
Characteristics exhibited by the radiating elements. (**a**) surface current distribution of the main radiating patch of the antenna at $\varepsilon _r$ = 2.5, 31.78 GHz. (**b**) equivalent circuit diagram (parasitic patch is unslotted).

All simulation results mentioned in the antenna design of this article were obtained through using HFSS (High-Frequency Structure Simulator), a software tool for electromagnetic simulation. Figure 3 exhibits the antenna return loss results derived from simulation under two different parasitic patches. As shown in Fig. 3a, when the dielectric constant of the LC material gradually increases from 2.3 to 3.3, the antenna’s center resonant frequency shifts from 32.38 GHz to 27.9 GHz while maintaining an impedance bandwidth of more than 9.05% when both parasitic patches are slotted. In contrast, Fig. 3b reveals that the operating frequency of the antenna shifts from 30.95-34.44 GHz to 26.68-29.19 GHz when they are unslotted, resulting in a reduction of the relative bandwidth from 10.67% to 8.99%. This indicates that the resonant frequency varies with the dielectric constant of the LC material, while the impedance bandwidth remains relatively stable. Combining Figs. 3a and 3b shows that the antenna can move from 33.46 GHz (31.92-35 GHz) to 27.39 GHz (25.39-28.86 GHz), yielding a reconfigurable frequency range of 18.14%. Moreover, the antenna maintains a stable frequency continuity controllable capability. The simulation results of the surface currents of the main radiating microstrip patch of the top dielectric plate of the antenna at $\varepsilon _r$ = 2.5 and an operating frequency of 31.78 GHz are shown in Fig. 4a, where the surface currents are stronger around the edges and slits of the radiating patch. The distribution of currents is not only confined to the middle region of the patch, but also appears in its two end regions, which indicates that the antenna excites multiple resonant modes and thus generates multiple resonant frequencies. As the path of the surface current increases, it causes the resonant frequency of the antenna to shift towards lower frequencies. An equivalent circuit model of the antenna radiation patch is shown in Fig. 4b. Guided by the equivalent circuit, the shape or size of the radiating patch is adjusted to affect its radiation performance at different frequencies.

**Fig. 5:**
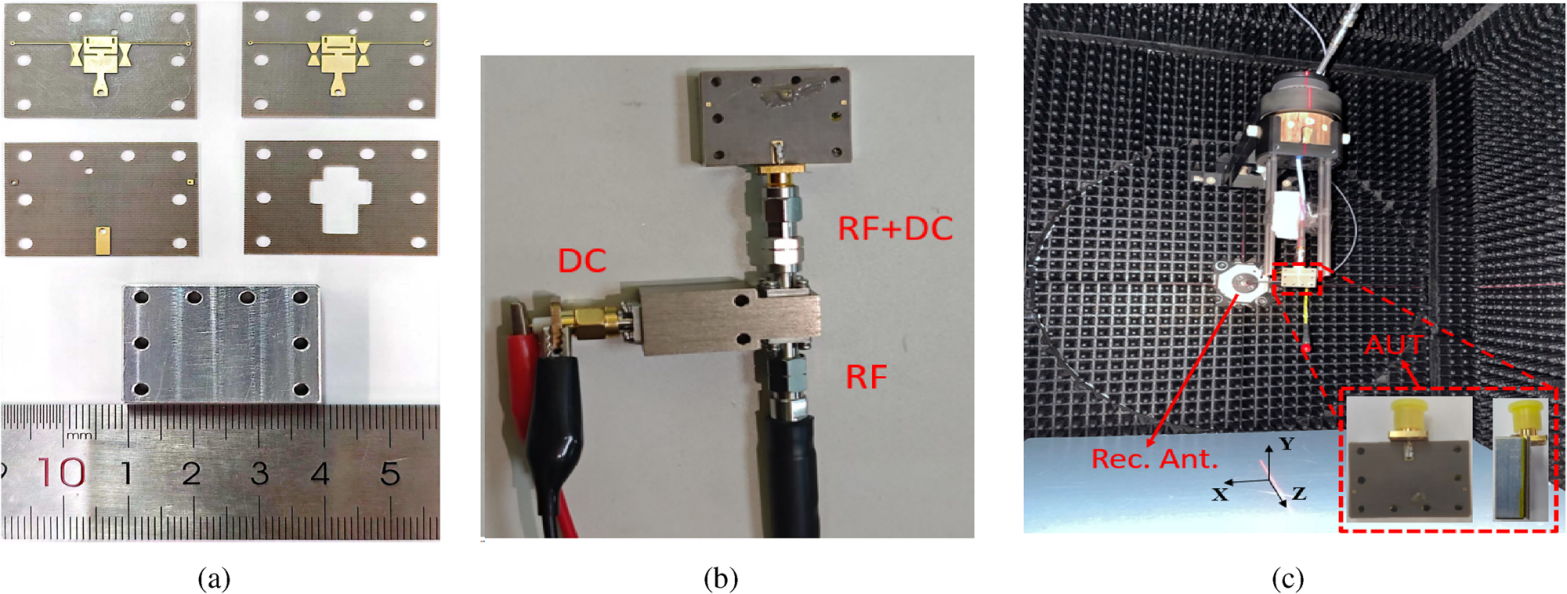
The actual processing and measurement diagram of the antenna is as follows: (**a**) physical objects on each layer of the antenna. (**b**) load dc-bias circuit for physical testing. (**c**) compact field range experimental environment (Rec. Ant. is represents receiving antenna).

To validate the proposed antenna design, the antenna prototype was actually tested using a Ceyear-3672D vector network analyzer (VNA) as well as a tight-field microwave anechoic chamber. The reflector and wave-absorbing materials of the anechoic chamber can concentrate and evenly distribute the electromagnetic waves radiated by the antenna under test (AUT) in the test area, in order to avoid errors in the test caused by external irregular electric fields. The test environment temperature is about $20^{\circ }$. Notably, the external bias voltage required to control the deflection of the LC’s molecules is applied to the electrodes through a T-shaped biasing device. This device prevents DC currents from flowing into the VNA, safeguarding the instrument from potential damage. This configuration ensures not only the accuracy of the test but also the safety of the operation. In addition, during the actual test, after changing the DC voltage value, it is necessary to wait for a period of time before testing. This is due to the fact that the electro-optical response of the LC will show hysteresis characteristic after the previous electric field is changed, i.e., the electric field triggered by the new voltage cannot be uniformly distributed immediately, which may lead to inconsistent alignment of the liquid crystal molecules, and thus lead to an unstable tuning mechanism. The physical images of each layer of the antenna are shown in Fig. 5a. Figure 5b presents the physical test image of the package loaded with T-type DC biasing device. The tight field physical test antenna environment image is shown in Fig. 5c.

**Fig. 6:**
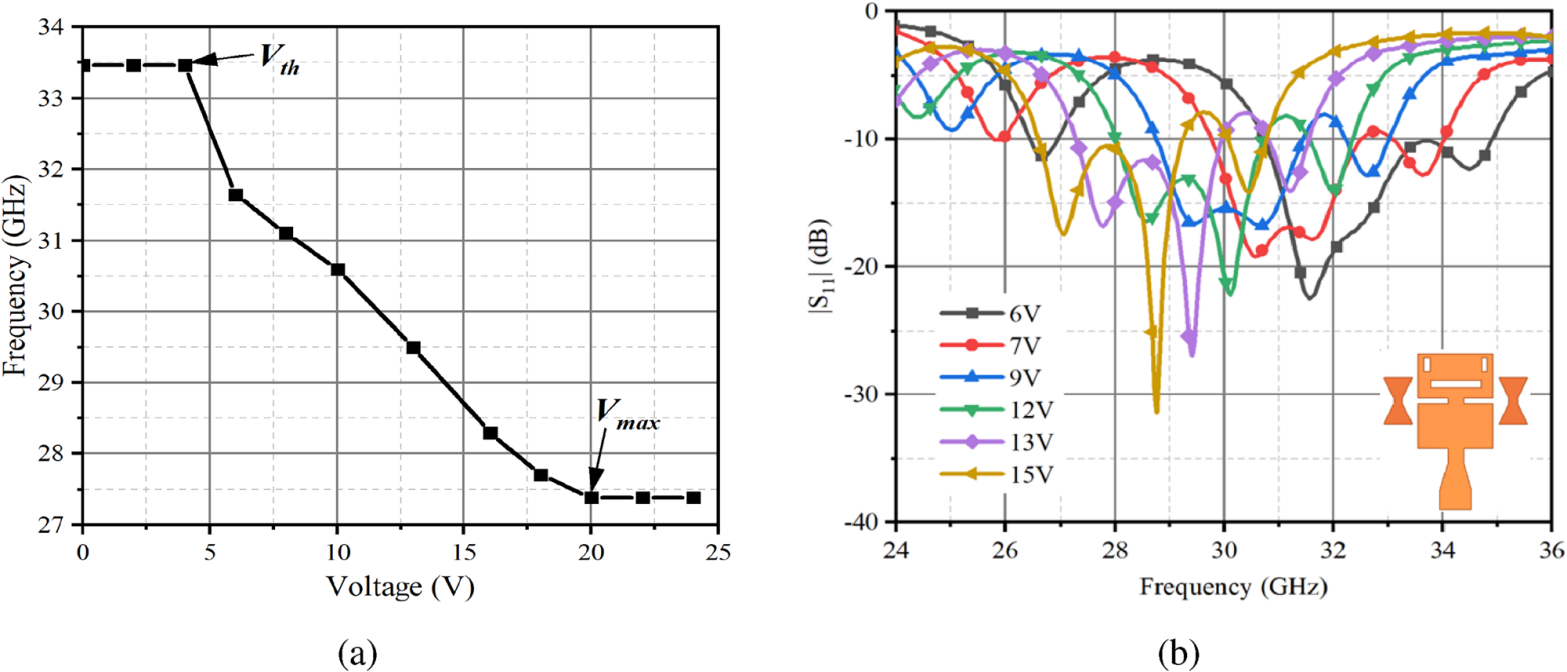
Measured antenna results with unslotted parasitic patch loaded. (**a**) the relationship between the resonant frequency of the reconfigurable antenna and the applied bias voltage. (**b**) measured resonance frequencies of the antenna at different bias voltages.

Figure 6 depicts the measured results of the antenna when the unslotted parasitic patches integrated. The antenna resonant frequency versus applied bias voltage is demonstrated in Fig. 6a. It is observed that that the operating central frequency of the antenna remains nearly constant at 33.46 GHz as the increase in voltage from 0V to 4.8V. However, beyond a voltage of 4.8V, the resonant frequency begins to gradually decrease. When the applied voltage reaches the saturation voltage of 20 V, the centre frequency of the antenna decreases to 27.39 GHz and thereafter the resonant frequency of the antenna remains stable without further shift as the applied voltage continues to increase. Figure 6b demonstrates the relationship between the applied different bias voltages and the operating resonant frequency of the antenna when the unslotted parasitic patch is measured. It is interesting to note that in Fig. 6b, compared with Fig. 3b, the antenna resonant frequency is shifted from 31.78 GHz to 27.1 GHz as the bias voltage increases, and the reconfigurable range of the resonant frequency reaches 14.73%. The overall agreement between simulated and measured results is in a good agreement, although some resonant frequencies exhibit slight low-frequency shifts. These deviations may stem from inaccuracies in processing and fabrication, such as the high temperatures used during polyimide curing, which may damage the orientation layer, or inconsistencies in the LC molecular distribution during encapsulation. In addition, air gaps within the stacked antenna structure may lead to a decrease in the dielectric constant of the dielectric layer.

**Fig. 7:**
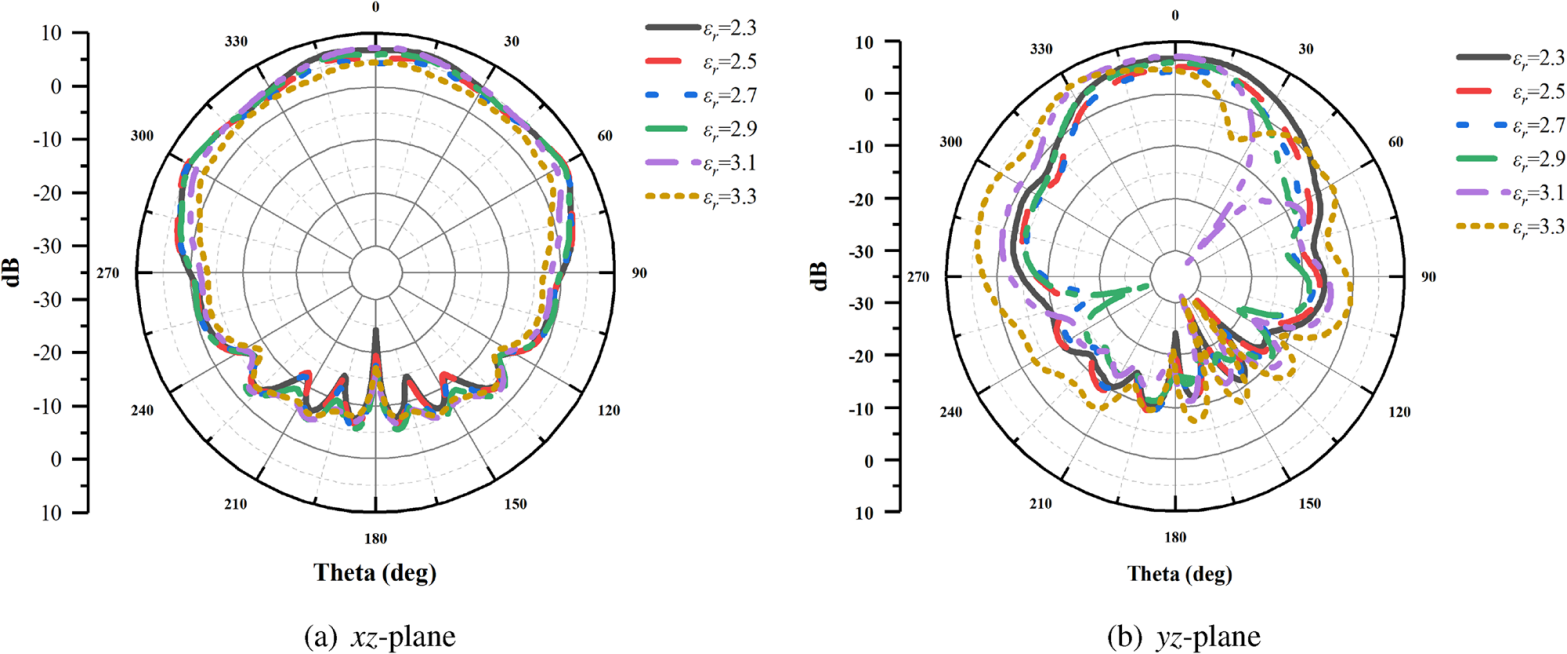
Radiation patterns of the proposed antenna on the (**a**) *xz*-plane and (**b**) *yz*-plane under different LC’s constant conditions and parasitic patches in the conductive state.

**Fig. 8:**
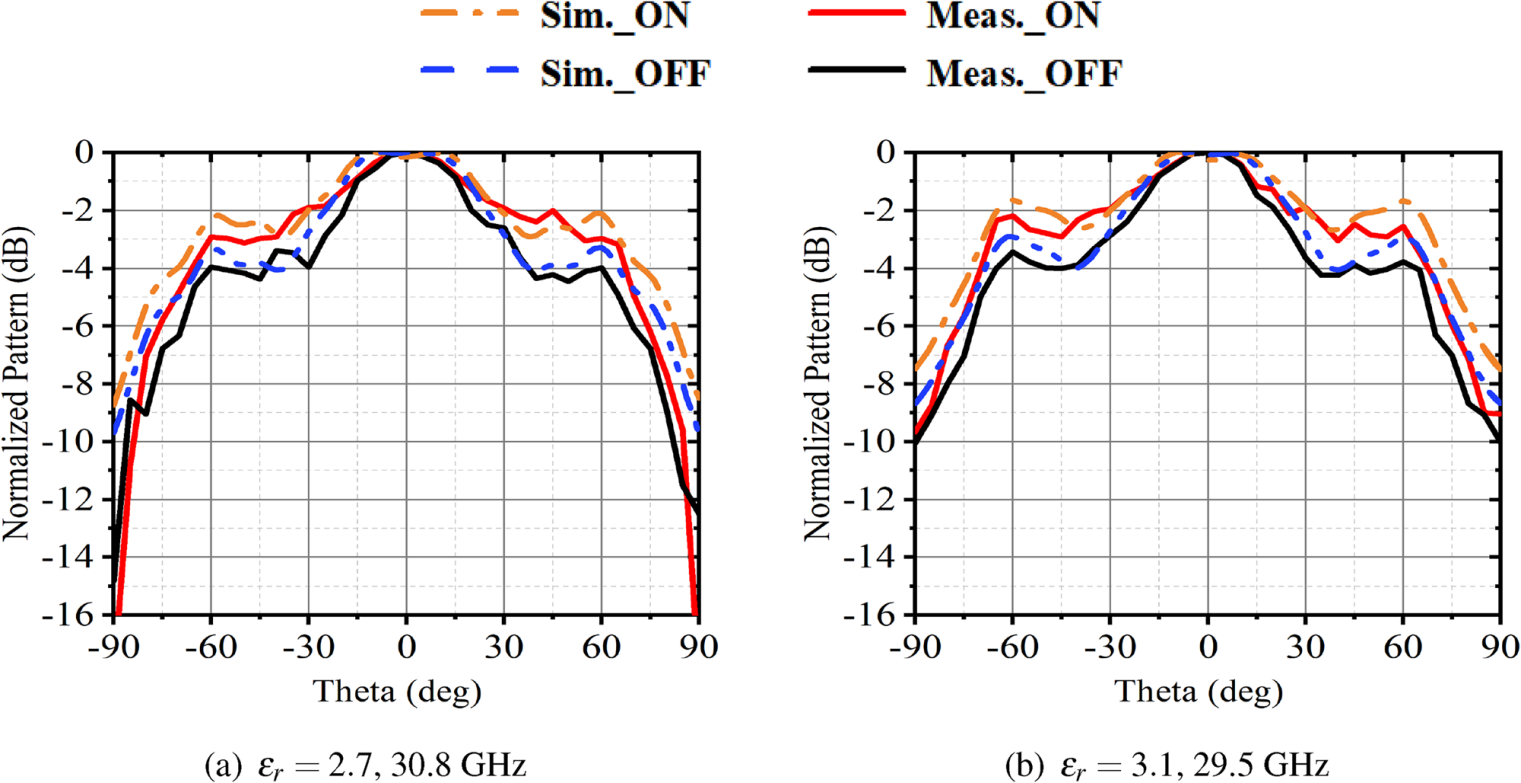
Normalized directional patterns of the *xz*-plane obtained from simulations and measurements of the proposed antenna under different states of parasitic patches in the conditions of (**a**) $\varepsilon _r = 2.7$, 30.8 GHz and (**b**) $\varepsilon _r = 3.1$, 29.5 GHz.

**Table 2:** Performance comparison between the proposed and other reported works. (N.A.U represents not applicable or unspecified).

		Reconfigurability				
Ref.	Tuning element	Fre. tunable range (GHz)	-3dB beamwidth tunable range (deg)	No. of feeding port	Antenna Configuration	Effective size ($\hbox {mm}^{2}$)
^11^	varactor diodes	2.15 - 2.99	50 - 58	1	Single antenna	$80\times 240$
^19^	LC	28.8 - 31.8	N.A.U	1	Single antenna	$12.9\times 12$
^22^	p-i-n diodes	0.79 - 0.96 1.71 - 5 and 27.5 - 28.35	N.A.U	4	Single antenna with Passive element	$34\times 8$
^23^	LC	19.5 - 27.7	N.A.U	1	Single antenna	$25\times 25$
^24^	p-i-n diodes	23.7 - 24.7 24.3 - 25.5 and 25.4 - 27.25 and 26.6 - 29.5	N.A.U	1	Antenna array	$34\times 32$
^25^	p-i-n diodes	26 - 30	N.A.U	1	Single antenna	$35\times 20$
Proposed	LC	27.1 - 31.78	53.5 - 81 and 47 - 92	1	Single antenna	$22\times 30$

Figure 7 illustrates the antenna radiation patterns in the *xz*-plane and *yz*-plane at 25.5 GHz obtained for various liquid crystal dielectric constants under slotted parasitic patch conditions. From the results in Fig. 7a, for dielectric constants of 2.3, 2.5, 2.7, 2.9, 3.1, and 3.3, peak gain of the antenna in the *xz*-plane is 7.59 dBi, 6.27 dBi, 6.04 dBi, 6.18 dBi, 7.58 dBi, 5.29 dBi, and in the *yz*-plane is7.48 dBi, 5.83 dBi, 4.43 dBi, 5.93 dBi, 7.58 dBi, 7.04 dBi, the half-power beamwidths remain nearly identical, with minimal variation observed in the radiation pattern in the *xz*-plane. This suggests that the antenna exhibits a better omnidirectional characteristic. Similarly, Fig. 7b demonstrates that the radiation performance in the *yz*-plane remains relatively uniform in all directions. Combined with the radiation pattern in the *xz*-plane and *yz*-plane, the adjustment of different dielectric constants has little effect on the average and peak gain of the antenna.

By comparing Figs. 8 and 3, it is evident that the resonant frequency and the radiation pattern of the antenna have a very low mutual coupling in the reconstruction. Specifically, while keeping the dielectric constant of the LC, the presence or absence of slots in the parasitic patch has a negligible impact on the antenna’s resonant frequency. Meanwhile, when the type of parasitic patch is fixed, adjusting the different dielectric constants of the LC have little effect on the shape of the radiation pattern and the -3dB beamwidth. Figure 8 presents the normalized pattern of the *xz*-plane obtained from simulations and real measurements of the antenna at different dielectric constants and operating frequencies. In Fig. 8a, the normalized radiation patterns are shown for the LC material with relative dielectric constant of $\varepsilon _r = 2.7$ and an operating frequency of 30.8 GHz when both parasitic patch structures are either disconnected or conducting. Figure 8b presents the normalized radiation pattern for $\varepsilon _r = 3.1$ and an operating frequency of 29.5 GHz. It is evident that the simulated and measured results align well for the same dielectric constant. When both parasitic patches are conducting, the -3dB beamwidth of the antenna increases from $47^{\circ }$ to $92^{\circ }$, resulting in an increase of 64.75% in the beamwidth. For $\varepsilon _r = 3.1$, the -3dB beamwidth of the antenna increases from $53.5^{\circ }$ to $81^{\circ }$, reflecting a beamwidth reconfigurable range of 40.89%. Table 2 summarizes the reconfigurable performance of the antenna presented in this study, comparing it with the results of related antenna designs documented in the literature.

## Conclusion

In this paper, an LC-based frequency and beamwidth reconfigurable antenna is proposed. By embedding the LC material into a separate dielectric layer, both frequency and beamwidth reconfigurability are achieved effectively. This design strategy effectively reduces the interference in the physical space, ensures the mutual independence between the two reconfigurable properties, thereby significantly reducing their mutual coupling effects. The impedance bandwidth of the antenna is successfully extended by employing an etched slot technique and optimizing the design of the parasitic dipoles. The dynamic control of frequency reconfigurability is achieved by exploiting the anisotropic properties of the LC material. Specifically, the bias electric field within the LC layer is regulated by adjusting the applied bias voltage, which in turn modulates the LC permittivity and thus frequency reconfigurability. This enables frequency tuning while maintaining a nearly constant -3dB beamwidth. Additionally, the radiation pattern’s beamwidth can be reconfigured at a fixed frequency by altering the slotting configuration at the junction of the parasitic patch arms. This modification, controlled by a specific dielectric constant, allows for a wide range of beamwidth reconfigurability. Unlike traditional mono-exponential reconfigurable antennas, this design overcomes their inherent limitations by maximizing spatial resource efficiency in communication systems. It meets the criteria for an integrated multifunctional antenna and minimizes the interactions between various reconfigurable functions. Consequently, the proposed antenna adapts to diverse communication needs, accommodating different regions and standards, enhancing simultaneous user access, and improving overall network performance. The design holds significant promise for applications in wireless communications, radar, remote sensing, and other advanced technologies.

## Data Availability

All data generated or analysed during this study are included in this published article.
